# On-Chip Reconfigurable Focusing through Low-Loss Phase Change Materials Based Metasurfaces

**DOI:** 10.3390/mi13122185

**Published:** 2022-12-09

**Authors:** Muhammad Shemyal Nisar, Shahid Iqbal, Linjie Zhou

**Affiliations:** 1 Sino-British College, University of Shanghai for Science and Technology, Shanghai 200093, China; 2 School of Electronics and information Engineering, Shenzhen University, Shenzhen 518060, China; 3 State Key Laboratory of Advanced Optical Communication Systems and Networks, Shanghai Key Lab of Navigation and Location Services, Shanghai Institute for Advanced Communication and Data Science, Department of Electronic Engineering, Shanghai Jiao Tong University, Shanghai 200240, China; 4 SJTU-Pinghu Institute of Intelligent Optoelectronics, Pinghu 314200, China

**Keywords:** low-loss phase change materials, integrated photonics, computing metasurfaces, reconfigurable metasurfaces

## Abstract

Metasurfaces are useful subwavelength structures that can be engineered to achieve useful functionality. While most metasurfaces are passive devices, Phase Change Materials can be utilized to make active metasurfaces that can have numerous applications. One such application is on-chip beam steering which is of vital utility for numerous applications that can potentially lead to analog computations and non-Von Neumann computational architectures. This paper presents through numerical simulations, a novel metasurface that can realize beam steering through active phase switching of in-planted arrays of phase change material, Sb${}_{2}$S${}_{3}$. For the purpose of numerical demonstration of the principle, beam focusing has been realized, on-chip, through active switching of the Sb${}_{2}$S${}_{3}$ unit cell between the amorphous and crystalline phases. The presented architecture can realize on-chip transformation optics, mathematical operations, and information processing, thus opening the gates for future technologies.

## 1. Introduction

Metasurfaces are those surfaces that have been artificially structured for the purpose of controlling and manipulating light through selective light-matter interaction [1,2]. This characteristic makes Metasurfaces an attractive platform for numerous applications including novel applications such as non-von Neumann computing architectures [3]. Metamaterial research has been the focus of researchers from a variety of disciplines because of numerous realized and potential applications for which they have proved to be extremely useful. These applications include imaging [4,5,6], cloaking [7,8], sensing [9,10], medical instrumentation [11,12], antennas [13,14,15], and data storage [16,17]. While most of the metamaterial platforms used free-space optics [18,19] and therefore were of limited utility for integrated applications, there has been a growing trend towards using phase change materials (PCMs) in order to make active devices.

Phase change materials are a unique class of materials that are extremely useful for a wide variety of applications due to their interesting material properties. These properties make PCMs important for the development of next-generation computing [17]. PCMs, exhibit multiple metastable states at room temperature. These different phase states have different electronic and optical properties [20]. The ability to easily reversibly switch between the states of PCMs enables re-programmability in the devices that use PCMs. Most of the re-programmable metamaterial devices employ PCM, such as GST [21], GSST [22], or VO${}_{2}$ [23], to realize active control of the electromagnetic waves and the re-programmability.

Although the PCM-based photonic circuits, have been popular and have found various applications [24,25,26,27], the applicability of these systems for applications such as beam steering is very limited. This is because conventional PCMs like GST and VO${}_{2}$ have very low figure-of-merit (FOM) at the desired wavelength of 1550 nm. The low FOM is due to very large optical loss as the imaginary part of the refractive index is large in crystalline as well as in amorphous state [17]. The lossy nature of PCMs leads to non-independent control of the phase and amplitude of the electromagnetic wave. Due to these problems in a GST-based platform, engineering of PCMs with high FOM, low-switching power, fast response times and good endurance is an ongoing research [28].

Recent developments in PCMs have presented Sb${}_{2}$S${}_{3}$ (henceforth referred to as, SbS) and Sb${}_{2}$Se${}_{3}$ [29], as promising low-loss PCMs which are theoretically lossless at the desired wavelength of 1550 nm. This enables independent control of the phase and amplitude of the optical wave and thus opening the way to reconfigurability.

**Figure 1:**
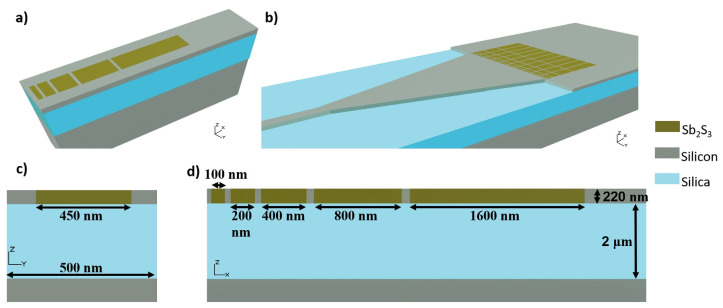
The schematic of the (**a**) single array of Sb${}_{2}$S${}_{3}$ cells of increasing length, (**b**) a photonic crystal composed of a number of arrays of Sb${}_{2}$S${}_{3}$ coupled with an incoming waveguide through a taper’s, (**c**) cross-sectional view of a unit cell and (**d**) a cross-sectional view of the array of Sb${}_{2}$S${}_{3}$ along with the measurements of each unit along the direction of propagation.

As the SbS is a theoretically lossless material [29], we present through numerical simulation a novel, silicon-based, active dielectric metasurface for on-chip reconfigurable focusing through the use of SbS. The structure of the photonic crystal used for the metasurface is given in Figure 1. The platform operates at the wavelength of 1550 nm and uses SbS, a phase change material, to achieve active switching. According to our knowledge, this is a first-ever implementation of such an active dielectric-based metasurface for on-chip beam steering.

## 2. Numerical Simulation Setup

The Finite Difference Time Domain (FDTD) method (using commercially available simulation software) is used for the numerical simulation of the metasurface. The model is made with a standard silicon wafer which has a silicon layer 220 nm thick and is set up in the environment of free space. The underlying layer of silica is 2 microns thick. Figure 1 shows the structure of a unit cell, an array of unit cells, the photonic crystal made from numerous arrays of unit cells, and their schematic. The distance between two neighboring arrays of unit cells is 100 nm.

The optical constants for Silicon and Silica are taken from Palik, the refractive indices used for SbS in the crystalline and amorphous states are 3.308 and 2.712 respectively [29]. Input is provided through a mode source of 1550 nm to a single-mode silicon waveguide and subsequently connected to the photonic crystal through a taper. To cater for the unwanted reflections, a perfectly matched layer (PML) is used as a boundary condition for all of the boundaries. To strike a balance between the accuracy of the results and the computational resources dedicated to the simulation, two different meshing regimes are deployed. A denser uniform mesh was deployed for the array of unit cells when simulating the array, and for the photonic crystal when simulating the whole metasurface. This denser mesh has a step size of 25 nm along the x-axis (the direction of arrays and that of propagation), 5 nm along the y-axis, and 25 nm along the z-axis. Whereas, the rest of the metasurface is meshed with a non-uniform mesh.

Initially, a single array is simulated to ascertain the behavior of the array under different material phase conditions of the unit cells in the array. As the length and width of an array are fixed and the lossless nature of the material implies that the loss in amplitude is minimal, the focusing can be realized through careful manipulation of the phase of the electromagnetic wave at the output. The change in phase (between crystalline and amorphous) of the phase change material brings about a change in the refractive index of that particular unit cell and thus the effective index of the complete array. This changes the phase of the output electromagnetic wave. Subsequently, several such arrays with different arrangements of refractive indices are arranged, into a photonic crystal, as shown in Figure 1, to realize the on-chip on-axis and off-axis focusing of the electromagnetic wave. While simulating the single array, to cater for unwanted reflection all of the boundaries were chosen to be PML and a denser mesh was created as described above.

## 3. Results and Discussion

A change in the effective refractive index of a section on the beam path can lead to a change in the phase of the transmitted beam. The different combinations of crystalline and amorphous unit cells cause a space-dependent phase shift in the incident beam along the direction of propagation. In the current case, the total effective refractive index depends upon the length of the array in the crystalline and amorphous phases.

Given that a single array of SbS has 5 elements which can have two different phases, this implies that an array can have $2^{5}$ = 32 different combinations for the arrangement of the refractive index. Each of these leads to a uniquely different phase of the output optical wave.

**Figure 2:**
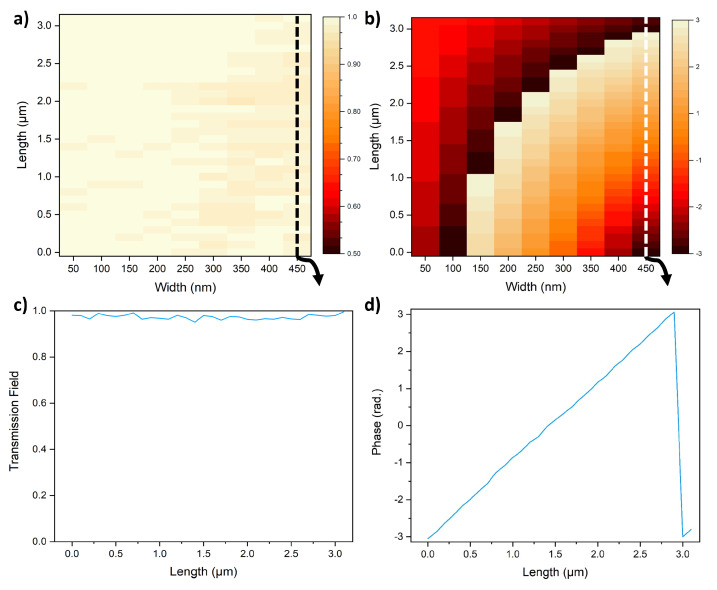
The distribution of transmitted amplitude and phase (**a**) Transmitted amplitude and (**b**) Transmitted phase as a function of length crystalline and the width of the array. (**c**) Transmitted amplitude and (**d**) Transmitted phase as a function of length for a fixed array width of 450 nm.

The phase shift along the direction of propagation can also be defined by Equation (1) [30], given below.

(1) $$\phi \left(y\right)=\frac{2\pi }{\lambda }n_{eff}\left(f-\sqrt{f^{2}+y^{2}}\right)$$

where λ is the operating wavelength, $\eta_{eff}$ is the effective refractive index and *f* is the focal length of the photonic metasurface. Therefore, an important way to look at the output from all of the different combinations of SbS array, from the perspective of device design, is as a function of an increasing length of crystalline SbS at the expense of the length of amorphous SbS. Given that each element of the array is twice in length compared to the previous element and the smallest element is 100 nm in length, using different combinations of crystalline and amorphous bits 32 unique lengths of crystalline SbS can be achieved with the difference of 100 nm between consecutive lengths. The transmitted amplitude and phase as a function of crystalline length and array width are shown in Figure 2a,b respectively. Figure 2c shows the transmitted amplitude for the array width of 450 nm. While the SbS itself is not a lossy material, the array structure nevertheless induces some loss through the reflection in cases where the refractive index variations are large. Considering the losses are not large, for the purpose of this work, they were largely ignored. The increasing length of crystalline SbS results in 32 uniquely different phases. As evident from Figure 2b, the phase variation increases by increasing the array width. This is because a larger width of the array allows the structure to interact more with the incoming electromagnetic wave. For the array with a width of 450 nm, the output optical wave shows phase coverage of 349°, as shown in Figure 2d.

As the phase coverage is extremely close to a full rotation of 360°, it shows that it is possible to achieve focusing using this array as a unit cell. But discretization of the output phase due to discrete lengths achievable through the platform will potentially impose limitations on the possible points of focus as well as the width of the focus point. Given the favorable phase coverage and no considerable losses, a slot width of 450 nm was chosen for this work.

**Figure 3:**
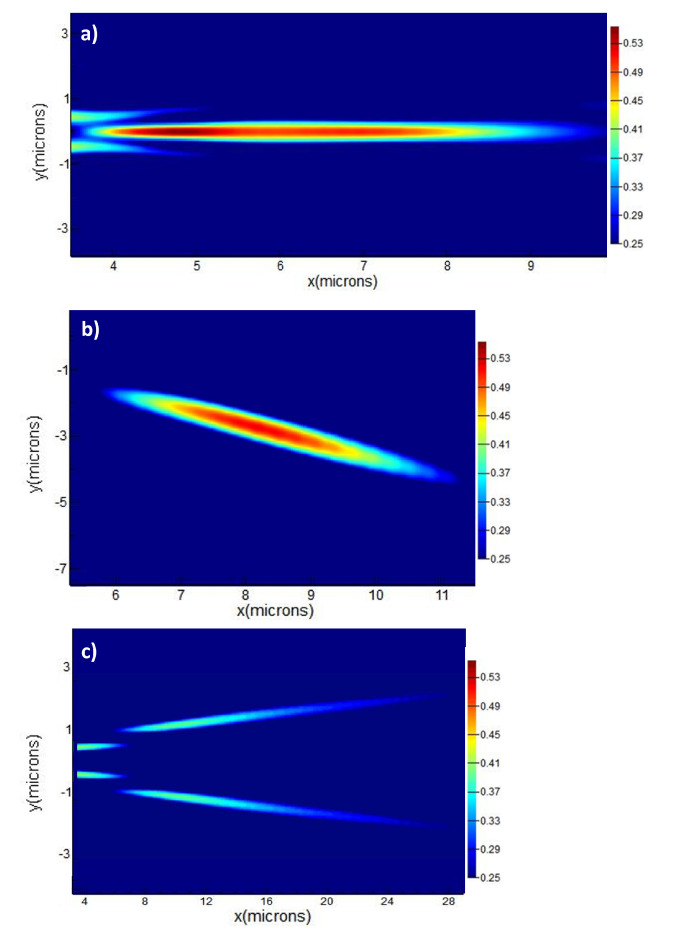
Simulated focusing achieved through the appropriate distribution of phases (**a**) On-axis focusing with increased depth of focus, (**b**) Off-axis focusing of the on-chip optical needle, and (**c**) beam splitting into two optical needles. The colorbar shows the electric field intensity and is kept the same for all of the figures so that to make the comparison easier.

Due to the quantized nature of the design of the device, it is not possible to achieve perfect phase matching at any point. Therefore, the material phase of SbS is arranged in a way to minimize the phase difference among the optical waves emanating from all of the different arrays of SbS. Through such an arrangement of material phase of SbS elements, beam focusing is achieved producing an on-chip optical needle as shown in Figure 3.

**Figure 4:**
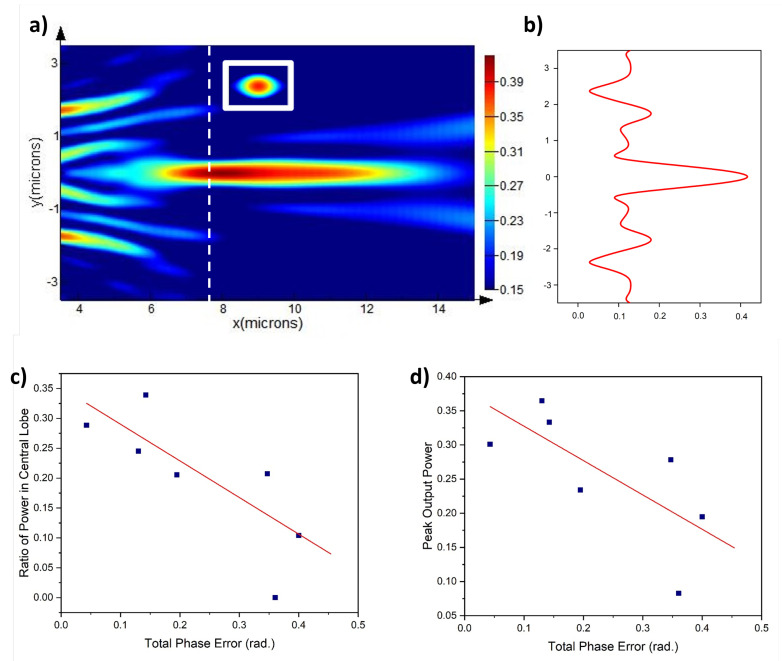
Central lobe and side lobes, as well as their relative power levels, are shown in (**a**) and (**b**) respectively. The inset shows the cross-section of the focal point. The relationship between total phase error and (**c**) ratio of power in the central lobe and (**d**) Peak output power.

Figure 3a show that the method achieved fairly reasonable focusing of the beam, while the programability entails that the point of focus and the depth of focus can also be changed according to the need. As the model presented uses quantized elements of SbS for focusing the beam, perfect alignment of phases from all of the SbS arrays is not possible. Although diffractive focusing always produces significant side lobes, the energy in the sidelobes achieved through this model will always be a little larger due to the quantized nature of the device architecture. This can be more clearly observed in Figure 4a,b. Furthermore, the ratio of power in the central lobe and the peak power also depend upon the total phase error of the photonic crystal. This relationship is vividly depicted in Figure 4c,d.

Figure 4c,d show that the ratio of power in the central lobe and the peak output power in the beam are inversely related with modulus of total phase error obtained by the phase arrangement in the photonic crystal. The total phase error is calculated by the summation of difference between desired phase at the focal point and the phase achieved at the focal point as given by Equation (2).

(2) $$TotalPhaseError=|\sum_{n=1}^{N}\left(\phi_{d}-\phi_{a}\right)|$$

Here, $\phi_{d}$ is the desired phase the focal point, $\phi_{a}$ is the phase achieved at the focal point, n is the array number representing the particular array in the photonic crystal and *N* is the total number of arrays in the photonic crystal. The actual total phase errors and the corresponding ratio of power in central lobe and peak power at focal point are plot given in Figure 4 along with best-fit lines. The Pearson’s correlation coefficients (PCC) for peak output power and ratio of power in the central lobe −0.8 and −0.9 respectively. These values of PCC show strong dependence between total phase error and the ratio of power in the central lobe as well as the peak power.

Beyond the on-axis focusing, the device was also explored for off-axis focusing and beam splitting. The results of this are shown in Figure 3c,d. The off-axis focusing can be achieved by choosing an off-axis point as the focal point and arranging the material phase of each unit cell in such a way that the resulting beam focuses at that particular point, while the beam splitting is achieved by constraining the material phase distribution in such a way that it is out phase at the center and gradually the phase from each unit cell is aligned as they move away from the central axis. Again, the quantized nature of the unit cell limits the number of points with a reasonably good focus, the device is nevertheless able to achieve reasonable off-axis focusing and beam splitting as shown in the Figure 3c,d respectively.

## 4. Conclusions

This article introduced a low-loss PCM-based platform for on-chip reconfigurable focus through numerical simulations. The PCM-enabled metasurface demonstrates on-chip focusing through active material phase switching between amorphous and crystalline material phases of SbS units in each unit cell. The presented results show that the SbS-based metasurface, due to the lossless nature of SbS, can be used to achieve programable on-chip focusing. But given the discrete nature of device design, it is bound to experience phase error at a lot of desired focus points. In light of this, the relationship of total phase error with the ratio of power in the central lobe and peak output power was calculated. The results show that the relationship between them is inverse which implies that with increasing phase error, the peak outpower and the ratio of power in the central lobe will decrease. This granularity in the device output can be reduced, but only at the expense of device size. The presented architecture can have potential applications in numerous fields such as on-chip transformation optics, mathematical operations, information processing, and optical logic gates.

## Data Availability

Not applicable.
